# The rites of spring, Take 2

**DOI:** 10.7554/eLife.16846

**Published:** 2016-05-18

**Authors:** Eve Marder

**Affiliations:** Department of Biology and the Volen National Center for Complex Systems, Brandeis University, Waltham, United States

**Keywords:** living science, grad school, careers in science, careers

## Abstract

Recruiting PhD students can be a frustrating process, but **Eve Marder** looks forward to welcoming the latest crop in the autumn.

In the spring of 1999, in an article titled “The rites of spring”, I wrote that I was depressed over the whole process of recruiting PhD students, with its promises, false and true, and its inevitable disappointments for applicants and programs alike (Marder, 1999). It is now 2016, and I am again involved in recruiting PhD students. The dismay I am feeling this year is acute, although as my mother used to say, we don’t remember pain very well, or women would never have a second baby. This year doing admissions has made me sufficiently miserable that it is hard to believe I have done this job about 25 times. I must forget this spring pain by the time that the new students arrive in the fall.

A lot has changed in the mechanics of how we recruit incoming PhD students over the past two decades, but much remains the same. In the old days all the applications lived in file folders color-coded by program in a drawer in the department office. The assistant who managed the files for our program did a wonderful job of sorting them into “invites”, “incompletes”, “admits”, “priority high holds”, “rejects” and so on. All I had to do was to go the office on a regular basis, pull out a pile of files, read them, scribble some notes, and then tell our assistant what actions to take. The files then magically migrated from one category in the file drawer to another, and it was always obvious what action was needed. All of the correspondence and emails were printed and put in the file, so any of us knew who had done what and when. Fast forward to 2016 and our admissions system is on-line, complete with a predictable array of associated inconveniences and improvements, including a surprising loss of transparency.

**Figure 1:**
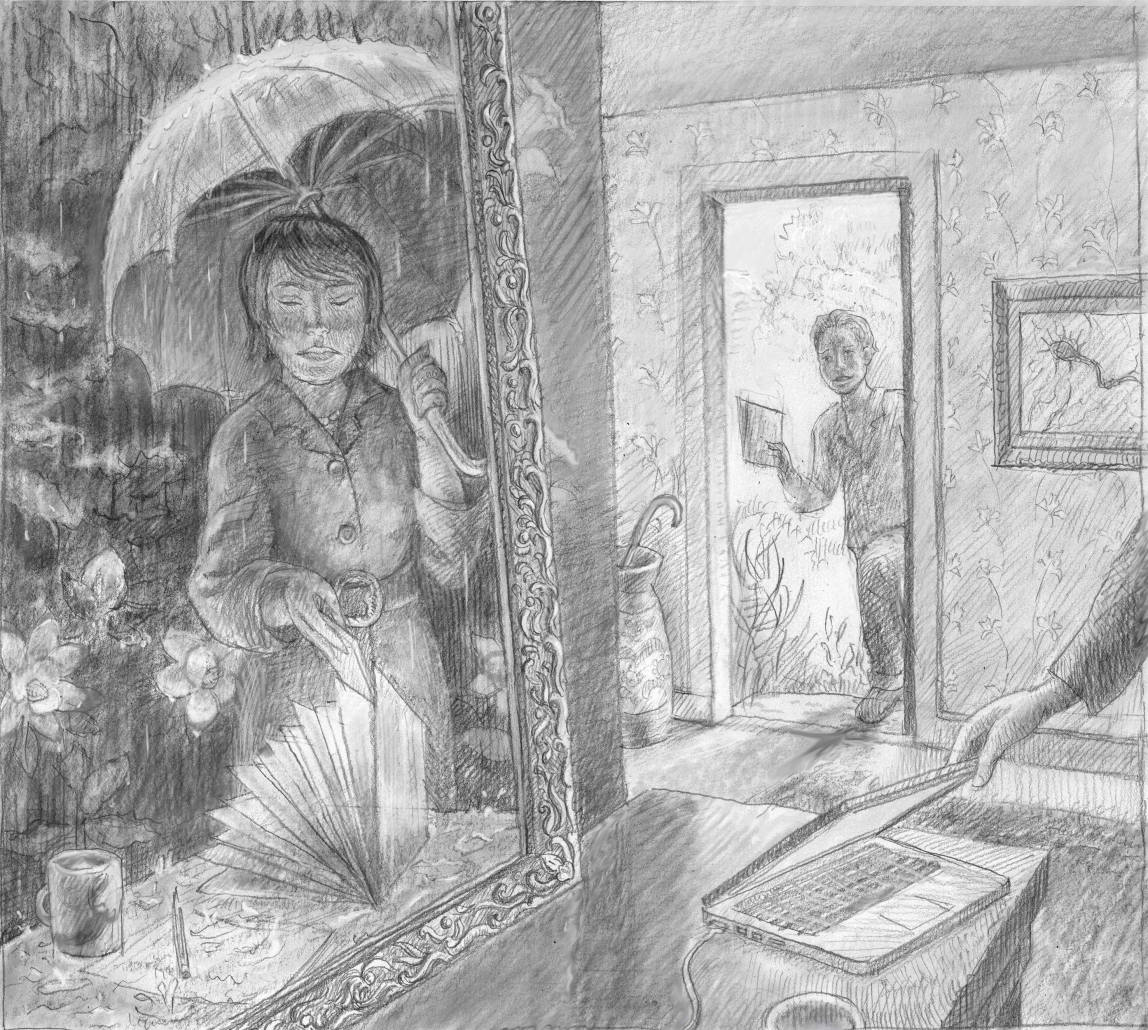
Many aspects of the recruitment of PhD students have changed over the past two decades, but the challenges of identifying those students who will thrive in a given program remain the same.

Illustration: Ben Marder

What hasn’t changed is my sadness at knowing that many aspiring scientists will be disappointed when they are not offered admission by the institution of their dreams. What hasn’t changed is my aggravation with colleagues who believe that we are making rational decisions about who is likely to become an outstanding scientist: the truth is that we are making facile decisions with no more reliability than if we were consulting a crystal ball. What hasn’t changed is my annoyance at applicants who are holding multiple offers and refuse to notify programs of their decisions in time so that we/they can make offers to other applicants. What hasn’t changed is my acute understanding that excellent paper credentials don’t always predict future success as a scientist, and some with much weaker records do have what it takes to be superb scientists. What continues to frustrate me, perhaps even more so today than in the past, is my sense that many applicants choose institutions where they are unlikely to flourish.

What has changed is the increasing number of male applicants who select programs on the basis of where their girlfriends are being accepted (we lost at least three excellent men this year because of 'two-body' issues, and that didn’t happen often 20 years ago). What has changed is the number of prospective students who are concerned about professional development, and are asking for programs to train them in skills they anticipate needing when they leave academic science. Some of today’s students are actively seeking information about opportunities in industry even before they start their PhD training. Unlike 20 years ago, today’s applicants share their impressions of programs and institutions via social media, so an applicant who has a great or a not great interview experience can influence other students, who might otherwise make a more independent decision on the basis of her or his own experience. What has changed is that we can now interview foreign applicants on Skype and get a much better sense of who they are and their interests, dreams and passions, than we could in the past.

In 1999 programs were actively courting applicants during the interview process with fancy dinners and excursions. But some time between then and now some programs have adopted “signing bonuses” which are substantial financial incentives to specific students. I profoundly dislike creating salary differentials between students who start PhDs at the same time, but other programs clearly don’t share these qualms. This reminds me of the signing bonuses paid by teams in the National Football League (NFL). Like the season for recruiting PhD students, the equivalent for American football – the NFL draft – rolls around each spring, with teams picking new players from the ranks of those moving from college football to the professional league. I wonder why my colleagues don’t worry more about the fact that many of the players picked in the first round of the draft do not become stars, or even make a career in the game, whereas many great players were picked in the later rounds of the draft: Tom Brady, for example, was a sixth-round pick and went on to become one of the most successful quarterbacks in the history of American football. If NFL teams, who employ full-time scouts to decide which players to pick, are unable to reliably identify the most promising football players, is it any wonder that we struggle to identify the prospective PhD students who will go on to become first-rate scientists?

What has changed is the increasing number of male applicants who select programs on the basis of where their girlfriends are being accepted.

As spring turns slowly to summer, I trust that my memory of this year’s spring admissions depression will fade. And I know that, come the fall, I will be pleased to greet a new crop of aspiring scientists. As a child I always waited with anticipation for the start of the school year, with new books, pristine notebooks and unknown subjects to learn. Although our incoming PhD students are far more sophisticated than we were many years ago, their excitement at starting a new era in their education helps remind us that science and education are precious and sacred callings.
